# Stereopsis and Eye Movement Abnormalities in Parkinson’s Disease and Their Clinical Implications

**DOI:** 10.3389/fnagi.2022.783773

**Published:** 2022-02-08

**Authors:** Fang Ba, Tina T. Sang, Wenjing He, Jaleh Fatehi, Emanuel Mostofi, Bin Zheng

**Affiliations:** ^1^Division of Neurology, Department of Medicine, University of Alberta, Edmonton, AB, Canada; ^2^Faculty of Science, University of Alberta, Edmonton, AB, Canada; ^3^Surgical Simulation Research Lab, Department of Surgery, University of Alberta, Edmonton, AB, Canada; ^4^Division of Neurology, Department of Medicine, University of Alberta, Edmonton, AB, Canada; ^5^Faculty of Medicine and Dentistry, University of Alberta, Edmonton, AB, Canada

**Keywords:** Parkinson’s disease, stereopsis, extraocular movements, biomarker, cognition

## Abstract

**Background:**

Parkinson’s disease (PD) is not exclusively a motor disorder. Among non-motor features, patients with PD possess sensory visual dysfunctions. Depth perception and oculomotor deficits can significantly impact patients’ motor performance. Stereopsis and eye behavioral study using 3D stimuli may help determine their implications in disease status.

**Objective:**

The objective of this study is to investigate stereopsis and eye movement abnormalities in PD with reliable tools and their correlation with indicators of PD severity. We hypothesize that patients with PD exhibit different eye behaviors and that these differences may correlate to the severity of motor symptoms and cognitive status.

**Methods:**

Control and PD participants were first evaluated for visual acuity, visual field, contrast acuity, and stereo perception with 2D and Titmus stereotests, followed by the assessment with a 3D active shutter system. Eye movement behaviors were assessed by a Tobii X2-60 eye tracker.

**Results:**

Screening visual tests did not reveal any differences between the PD and control groups. With the 3D active shutter system, the PD group demonstrated significantly worse stereopsis. The preserved cognitive function was correlated to a more intact stereo function. Patients with PD had longer visual response times, with a higher number of fixations and bigger saccade amplitude, suggesting fixation stabilization difficulties. Such changes showed a positive correlation with the severity of motor symptoms and a negative correlation with normal cognitive status.

**Conclusion:**

We assessed stereopsis with a 3D active shutter system and oculomotor behaviors with the Tobii eye tracker. Patients with PD exhibit poorer stereopsis and impaired oculomotor behaviors during response time. These deficits were correlated with PD motor and cognitive status. The visual parameters may potentially serve as the clinical biomarkers for PD.

## Introduction

Along with the cardinal motor symptoms of Parkinson’s disease (PD), patients with PD possess sensory visual and oculomotor dysfunctions. Visual signs and symptoms of PD may include defects in extraocular movement (Pinkhardt and Kassubek, 2011), pupillary function (Micieli et al., 1991; Wang et al., 2016), and higher-level complex visual tasks (Armstrong, 2017). Among these dysfunctions, impairment in depth perception–namely the ability to judge distance, the shape of an object, and the speed of movements–can significantly impact patients’ motor performance when impaired. It was reported that patients with PD yield more errors when making 3D judgments (Trick et al., 1994; Maschke et al., 2006; Kim et al., 2011). Patients with PD were notably impaired in spatial orientation (left–right and front–back), immediate visual-spatial recognition memory for mirror image patterns, 3D constructional skills, visual-spatial attention, and 3D mental rotation (Natsopoulos et al., 1993). Multiple factors in the central and peripheral visual pathway may work congruently in contributing to the pathology of depth perception in PD.

Difficulties in navigation due to stereopsis impairments can make patients with PD more susceptible to falls and injuries (Armstrong, 2011, 2017) and might contribute to changes in walking speed (Young et al., 2010). It has been noted that patients with PD with abnormal stereopsis performed significantly worse in motor function tests than those with normal stereopsis (Kim et al., 2011; Sun et al., 2014). The Hoehn and Yahr (H&Y) stages and Unified PD Rating Scale motor scores (UPDRS-III) were higher in PD patients with abnormal stereopsis than those with normal stereopsis (Sun et al., 2014), suggesting a coupling between stereopsis and motor performance in PD, or the association of depth perception difficulties with the progression of PD. In addition, patients with PD with freezing of gait (FOG) had worse stereopsis than those without FOG (Alhassan et al., 2020). However, there is limited knowledge on how depth perception in patients with PD changes as the disease progresses. Some evidence has also indicated that patients with drug naïve PD showed decreased stereopsis, raising the question that this specific visual change might be an early disease indicator (Kim et al., 2011).

The motor symptoms of PD can be treated using dopamine replacement therapy or surgical interventions, i.e., deep brain stimulation. Strong literature is still lacking for medical and surgical treatment of non-motor symptoms of PD, including visual sensory impairments. Sometimes, PD treatments can also cause visual side effects (Armstrong, 2011) notably visual hallucinations. On the other hand, it has been observed that subthalamic deep brain stimulation can improve eye movement abnormality, namely smooth pursuit and saccade (Fawcett et al., 2010; Yugeta et al., 2010; Nilsson et al., 2013; Antoniades et al., 2014). Taken together, stereopsis and eye movement behaviors can be further studied to determine their clinical implications in disease status.

To date, there is scarce scientific research on the stereoscopic functions in patients with PD due to limitations in methodologies and the lack of prospective observations. The widely used Titmus Stereotest and Random-Dot Stereogram showed some evidence of abnormal stereopsis in a group of older PD patients (68.6 ± 8.98 years) with a poor cognitive function (Kim et al., 2011). However, one should note that these stereotests assess binocular disparity without assessing the monocular function. Although the stereopsis is the perception of depth created by binocular cues, with horizontal disparities between the two retinal images being the primary cue for depth, any domain of the monocular peripheral pathways, such as acuity, color, contrast, relative size, texture gradients, and motion parallax, may impact 3D vision during abnormal. Without confirming a relative intact afferent monocular function, stereoscopic assessment is unreliable.

Several studies compared the validity, reliability, and effectiveness of different stereo tests, i.e., Random-Dot Stereogram, Titmus, Frisby, Lang II, and the conclusion was varied (Garmham and Sloper, 2006; Heron and Lages, 2012; Westheimer, 2013; Momeni-Moghadam et al., 2021). As a screening test, random-dot stereograms may not be the best choice to evaluate stereo blindness as correct perception not only requires normal stereopsis but also other higher cortical processes. The test does not differentiate between peripheral and central deficits. For these reasons, software-based stereo tests were established, which could be installable and adaptable to different possible visualization setups and devices (Gadia et al., 2014). Such a test was designed to test stereo acuity and blindness using the same visualization conditions and setup adopted for the tasks to perform, as well as offering better control of parameters like luminance levels. The test can be performed on a 3D TV display based on active shutter systems to produce depth perception. This method of depth perception evaluation is distinct from conventional stereo tests as the shutter system is actually optical rather than mechanical when it comes to forming 3D imaging. Another advantage of using 3D TV systems as a stereo measurement tool is that it allows participants to be tested from different distances, e.g., from the presented stimuli on the screen, and based on the results of the software-based test, stereo acuity can be calculated.

To form 3D images, both eyes receive slightly different images of a visual scene. Therefore, the adjustment aligning the two eyes is necessary to reconcile this disparity and achieve a cohesive 3D vision. To assess stereopsis, eye movements should also be taken into consideration. Using eye movement behaviors, i.e., fixation patterns as an index, they may provide insights into 3D perception as well as motor performance in PD. Some previous studies have shown that eye movement patterns are sensitive to 3D shapes (Vishwanath and Kowler, 2004; Wexler and Ouarti, 2008). In PD, saccade and vergence eye movements are reported to be abnormal (Gupta et al., 2021)–e.g., patients with PD experience increased reaction time in direction errors and slow saccade (Chan et al., 2005; Perkins et al., 2021), interfering with the perception of depth and dimensionality critical for normal balance control and the prevention of falls (Urwyler et al., 2014). Eye-tracking technology provides a simple and non-invasive yet effective way to assess the oculomotion that reflects the brain function (Sweeney et al., 1998; Tseng et al., 2013).

The objectives of the current study are to investigate the stereo function in PD using 3D active shutter visualization technology, taking the monocular cues into consideration when testing stereopsis, as well as investigating the oculomotor function. We propose that with the software-based system using 3D TV, even subtler deficits of stereo perception in PD can be detected. In addition, with the eye-tracking technology capturing patients’ eye response to stimuli, we aim to reveal a visual searching strategy. We hypothesize that patients with PD may exhibit a poor stereo function and different eye movement behaviors, and the types and degree of such changes may correlate to the severity of motor symptoms and certain non-motor symptoms, such as cognitive function.

## Materials and Methods

Patients with PD aged 40–70 years old and with H&Y stage one to four were recruited from the Parkinson and Movement Disorders Program (PMDP) at the University of Alberta along with age- and sex-matched healthy controls (HCs). Community volunteers without neurological disorders were recruited as HCs. About 20 patients with PD and 24 HCs participated in this study (Table 1). All patients with PD were assessed at PMDP and the Surgical Simulation Research Lab at the University of Alberta. PD subjects were assessed by movement disorder neurologists, and the diagnosis of idiopathic PD was based on UK PD Society Brain Bank criteria (Hughes et al., 2002). Patients with PD were optimally treated with PD medications. The study was approved by the University of Alberta Ethics Review Board, and all participants provided written informed consent prior to participation. The exclusion criteria were atypical or secondary parkinsonism, confounding medical or psychiatric condition(s), dementia or any condition that prevents patients from signing consent, other neurological diseases leading to motor dysfunction, and visual acuity of at least 20/40 in either eye.

**TABLE 1:** Demographic and clinical characteristics of patients with Parkinson’s disease (PD) (*n* = 62).

Clinical characteristics	Control (24)	Parkinson’s Patients (20)	*P*-value
Age (years)	62.2 ± 7.5	66.8 ± 8.4	0.059
Sex (M:F)	13:11	11:9	>0.25
Disease course (years)	N/A	9.8 ± 4.7	
UPDRS-III	N/A	18.9 ± 8.2	
LEDD (mg)	N/A	1219.7 ± 719.9	
MoCA	27.8 ± 1.8	25.8 ± 2.8	0.10
Visual Acuity (logMAR)			
OD	0.11 ± 0.13	0.16 ± 0.12	0.08
OS	0.11 ± 0.12	0.11 ± 0.12	>0.25
OU	0.04 ± 0.14	0.05 ± 0.13	>0.25
Contrast acuity (Pelli-Robson log unit)	2.0	2.0	>0.25
Titmus stereotest (seconds of arc)	47.0 ± 25.6	58.4 ± 24.7	>0.25
3D TV Stereo acuity (seconds of arc)	40.1 ± 29.3	74.1 ± 35.7	0.005*

M, male; F, female; UPDRS-III, Unified Parkinson’s Disease Rating Scale-Part III; LEDD, levodopa equivalent daily dose; MoCA, Montreal Cognitive Assessment; OD, oculus dextrus; OS, oculus sinister; OU, oculus uterque. *p < 0.05.

### Clinical Assessments

All participated patients with PD were evaluated for their motor symptoms using the UPDRS-III and H&Y stage. Medication usage summarized as levodopa equivalent daily dose (LEDD) (Tomlinson et al., 2010) for each patient is shown in Table 1. At the study enrollment, other demographic and clinical information, i.e., educational level, duration of the disease, and cognitive testing with Montreal Cognitive assessment (MoCA), were obtained by a movement disorder neurologist or the research coordinator.

### Visual and Stereo Screening and Stereo Acuity Tests

Visual acuity was tested using the Snellen chart. Corrected visual acuity was recorded for each eye followed by binocular vision. Contrast sensitivity was performed using the Pelli-Robson test. People with abnormal visual acuity (worse than 20/40) and contrast sensitivity were excluded as these abnormalities may represent optic nerve or retina pathologies, as well as media opacities and macular disease that may skew the stereo perception results. Screening stereopsis was performed with 2D pictures demonstrating the depth estimation from image structure, which was based on whole scene structures that do not rely on specific objects (Li et al., 2006). With 2D images, study participants were asked to estimate the depth relationship in each picture. Using three pictures, we show two or three objects of uniform size that are distributed in depth (Supplementary Figure 1). The closest object will project to a retinal image with the largest angular size. The other farther objects projected to retinal images are relatively smaller. The study participants were asked to provide a cue to the depth judging the relative sizes of the objects. If the mistakes were made, the participants will be excluded from further assessment. None misjudged depth with the 2D pictures. In addition, the standard and widely used Titmus stereotest (Stereo Optical Co., Inc., Chicago, IL, United States) was utilized. These tests can reveal stereo perception difficulties as well as compare and validate the results from the 3D monitor active shutter system.

### Stereo Acuity Function Test With 3D Active Shutter System

**FIGURE 1:**
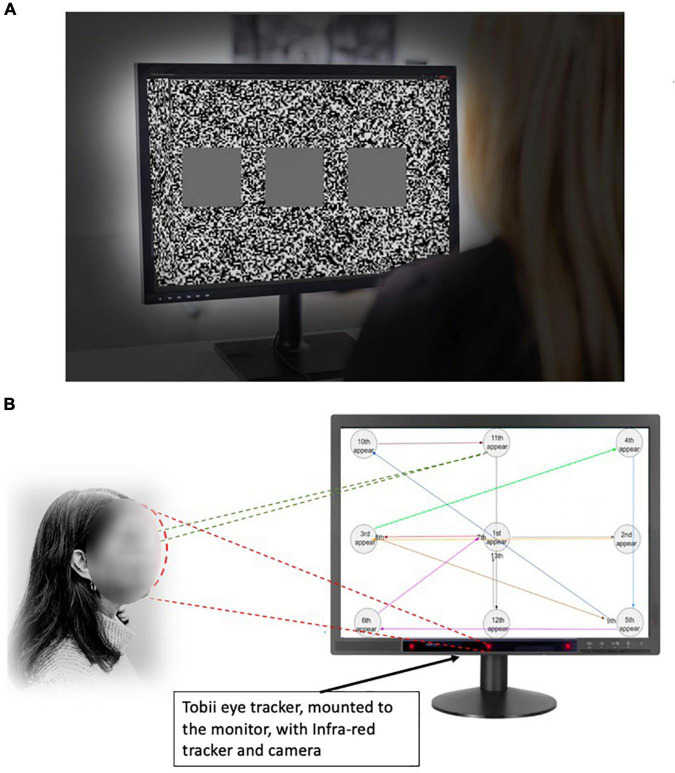
Experimental settings to evaluate stereopsis with the 3D monitor active shutter system. **(A)** The 3D monitor active shutter system. The visual stimuli were presented on a 3D monitor using a 24-inch 3D video monitor. A special pair of shutter glasses containing a liquid crystal layer was used. The images were set at a refresh rate of 60 frames/s. The visual stimuli for each testing position consisted of a set of three squares, placed side-by-side placed in the foreground with a fixed white noise background. One of the squares, randomly decided, was of a one-pixel disparity while the others are presented at zero parallax. **(B)** The eye-tracking system. The Tobii eye tracker was installed beneath the 3D TV monitor. The gaze “hot spots” and position data can be extracted from the TV watching. The participants were asked to track a moving object with their gaze as it appeared on the screen at 13 positions in a predetermined order, and the speed of appearing/disappearing of the visual object remained the same.

After the screening tests, study participants were examined for stereoacuity functions. The visual stimuli were presented on a 3D monitor active shutter system. A 24-inch 3D video monitor (Tobii 1750 LCD Monitor, Tobii Technology, Stockholm, Sweden) was used for a test (Figure 1A). The active shutter system uses a special pair of shutter glasses containing a liquid crystal layer. The images were set at a refresh rate of 60 frames/s. We were able to perform the assessment of stereo perception of the participant directly on the device they would use for the stereoscopic working task. The display has a resolution of 2,470 × 1,240 pixels, at a 60 Hz refresh rate. It is equipped with passive stereoscopic visualization based on circularly polarized filters. The pixel display is equal to or similar to most of the standard desktop monitors. Knowing the distance and the angle of view between the screen and the participant, the disparity among the test images can be calculated (Gadia et al., 2014). The test was performed with the lights off. In this phase, the participants were asked to complete a visual task while seated in eight different positions, varying in the distance (0.7/1.4/2.1/2.8 m) and viewing angle (45° and 90°) (Gadia et al., 2014). The task entailed a sequence of 10 visual stimuli. Each test was consisted of a set of three squares, placed side-by-side placed in the foreground with a fixed background (a white noise random-dots pattern). One of the squares, randomly decided, was of a one-pixel disparity while the others are presented at zero parallax. The participant must verbally report which square was perceived in front of the others. Black background for 2 s was shown before the next test image. The order of the subject’s position and the order of the stimuli presented were counterbalanced. The accuracy of the stereo perception was calculated and recorded. The stereo acuity corresponding to the positions are 160, 100, 63, 50, 40, 32, 26, and 20 arc seconds, respectively (Eidolab, 2014). The participants were instructed to not guess and to report the absence of disparity according to their perception. We analyzed the stereo acuity in two ways. First, for the absolute acuity, for any given set of 10 stimuli, if the accuracy was less than 70%, the stereo acuity was assigned to the previously tested level. The choice of using 10 stimuli was to minimize the chance of guessing. Second, we used the percent accuracy for each participant at a given acuity level from 160 to 12.5 arc seconds. Another method used in the current study was to utilize the percentage accuracy at each tested arc second to compare which method is more sensitive to detect stereo abnormalities. The normal stereo acuity was deemed to be between 32 and 63 arc seconds. The choice of using percent accuracy was to determine whether it is a more sensitive measure, as in the methods described by Gadia et al., they observed substantial equivalence of the two tests, down to the disparity of 26 arc seconds. From 20 to 12.5 arc seconds, the software-based test detects more abnormalities (Gadia et al., 2014).

### Oculomotion Patterns

We evaluated the eye motion pattern to reveal specific eye behaviors, namely saccades and fixations in patients with PD. The Tobii X2-60 eye tracker (Tobii Technology, Inc., Danderyd, Sweden) was installed beneath the 3D TV to record subjects’ eye movements. Equipped with the two hidden IR cameras, Tobii X2-60 captured gaze location over the TV screen at 60 Hz after calibration. The choice of eye tracker at 60 Hz is constrained by the simultaneous use of a stereoscopic display in our study. The reported resolution of Tobii X2-60 was 0.5 visual degrees when the eye tracker was placed at an ideal distance from the participant. The built-in software allowed the synchronization and analysis of the gaze data (eye motion trajectory) when the images were shown on the screen.

To perform the eye tracking, participants were asked to track the moving object with their gaze as it appeared on the screen at 13 positions in a predetermined order. The speed of the appearing and disappearing visual object remained the same. During the entire trial, participant’s eye movements were recorded by the Tobii X2-60 eye tracker (Figure 1B). The visual response time was measured on each object’s appearance, calculating from the time when the target appeared to the moment when the eyes gazed upon it.

### Statistical Analysis

The differences between patients with PD and HCs in a visual and contrast acuity, visual field, stereo screening with the 2D pictures and Titmus Stereotest, stereo acuity using the 3D TV shutter system, and the eye-tracking measures were compared using the Mann–Whitney *U*-test. The correlation between stereopsis and eye-tracking parameters with UPDRS motor scores, LEDD, course of the disease, and MoCA were evaluated using univariate regression models. Standardized estimated regression coefficients (β) and the coefficients of multiple determination (*R*) were calculated. *p* < 0.05 was considered as significant.

## Results

**FIGURE 2:**
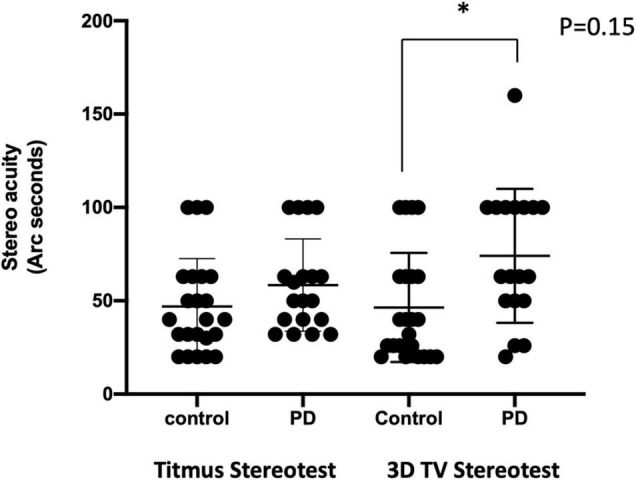
Comparison of the stereo acuity using different methods. Stereo acuity (acuity in arc seconds) assessment with Titmus stereotest was shown on the left, and the 3D active shutter system was shown on the right. *, *p* = 0.15, with Mann–Whitney *U*-test.

Between the control and PD groups, patients with PD were 5.6 years older. Among the 20 patients with PD recruited (Table 1), 45% had a moderate disease with an H&Y ≥III. The mean LEDD was 1,220 ± 720 mg, with a disease duration of 10 ± 5 years at the time of assessment. Patients with PD had a lower MoCA score than control but they were not statistically significant. There were no differences in visual acuity (OD, *p* = 0.41; OA, *p* = 0.88; OU, *p* = 0.30), visual field (*p* > 0.99), and contrast acuity (*p* > 0.99). With the stereo acuity between 32 and 63 arc seconds being considered normal, the stereopsis with the 2D pictures and the Titmus stereotest revealed normal results in both groups (Figure 2).

### Stereo Acuity Function Test With the 3D Monitor Active Shutter System

**FIGURE 3:**
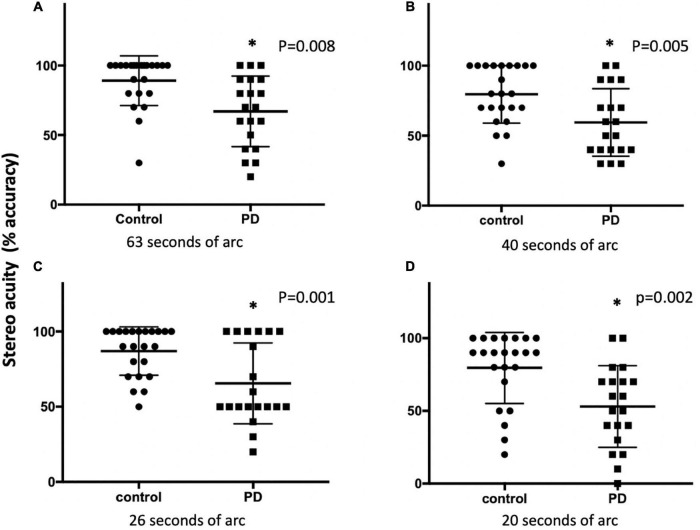
The stereo acuity between the control and PD groups using the 3D active shutter system at 63 **(A)**, 50 **(B)**, 40 **(C)**, and 26 **(D)** arc seconds. The stereopsis was presented as percent accuracy. *p < 0.05, with Mann–Whitney *U*-test. PD, Parkinson’s disease.

**FIGURE 4:**
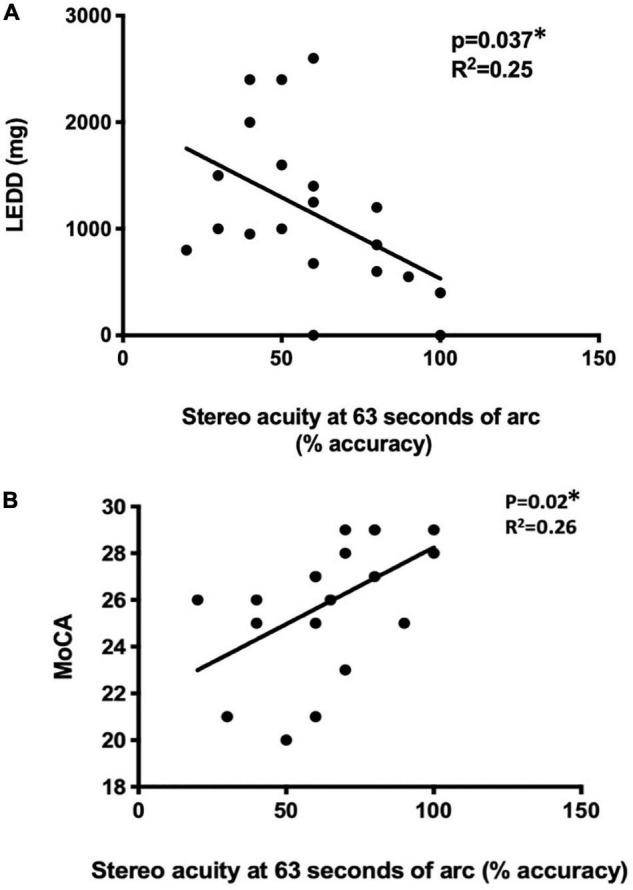
The correlation of disease status with stereo acuity. Disease severity was reflected by LEDD **(A)** and cognitive function was assessed MoCA **(B)**. *p < 0.05, using univariate regression models. LEDD, levodopa equivalent daily dose (mg), MoCA, Montreal Cognitive Assessment.

With the 3D active shutter system measuring absolute acuity, the PD group had a significantly worse stereo acuity (74 ± 36 arc seconds) when compared with the control group (40 ± 29, *p* = 0.005, Figure 2). Patients with PD performed the best at the most optimal position—0.7 m right in front of the monitor (160 arc seconds). With the percent accuracy method, the 3D active shutter system was clearly more sensitive in detecting the deficit in stereopsis compared to the Titmus stereotest. It is notable that even at 160 arc seconds, 45% of patients with PD were already making mistakes in their 3D perception, while only 16.7% of control individuals misjudged the disparity. We observed significant changes in stereo perception starting at 63 arc seconds in patients with PD judging the percent accuracy (Figures 3A–D, *p* < 0.05). LEDD was negatively correlated with the stereo acuity (*r* = −0.5, *p* = 0.037, Figure 4A), and there was a trend that UPDRS-III was negatively correlated with the stereo acuity (*r* = −0.42, *p* = 0.067). However, disease course on its own did not have any correlation with stereo acuity. In addition, in patients with PD, a preserved cognitive function (near normal MoCA score) was linked to a more intact stereo acuity (*r* = 0.51, *p* = 0.015) (Figure 4B). These observations suggested that poorer stereo perception can be related to disease severity. These correlations are representative of what holds for the levels of disparity between 63 and 20 arc seconds.

Using the absolute stereo acuity measured with the 3D active shutter system, no correlation was observed between the acuity with the UPDRS-III score, LEDD, duration of the disease, or MoCA. There was no correlation detected between the stereo acuity measured with the Titmus stereotest with the clinical features in the PD group.

### Eye-Tracking Analysis

Using the Tobii eye tacking system, the response time of the PD group (444 ± 73 ms) was significantly longer than that of the control group (409 ± 62 ms, *p* = 0.014).

**FIGURE 5:**
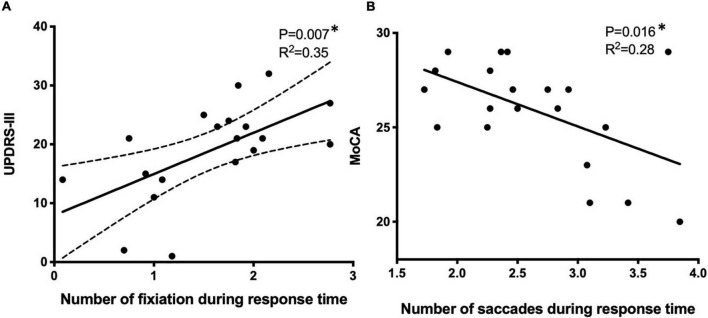
The correlation of eye motion trajectory with disease status using the univariate linear regression model. Eye motion trajectory data were analyzed against UPDRS-III **(A)** and MoCA **(B)**. *p < 0.05, using univariate regression models. UPDRS-III, Unified Parkinson’s Disease Rating Scale, MoCA, Montreal Cognitive Assessment.

Although patients with PD tended to perform worse, no significant differences were observed in the total number of fixations (17 ± 9 vs. 19 ± 8), average fixation duration (274 ± 124 vs. 280 ± 107 ms), average saccade amplitude (7.4° ± 3.2° vs.8.2° ± 3.3°), or the total number of saccades (31 ± 8 vs. 34 ± 9) between control and PD groups. However, when analyzing the response time between the target disappearing and the next target appearing, the number of fixations was higher (1.4 ± 0.9 vs.1.5 ± 1.1, *p* < 0.01) in the PD group, suggesting that patients with PD expressed difficulties in keeping their fixation on the fixation point when compared with the control group. The number of fixations during response time was correlated with the severity of motor symptoms as reflected by UPDRS-III (Figure 5A, *p* = 0.007, *r*^2^ = 0.35), while the number of saccades during response time was correlated with MoCA (Figure 5B, *p* = 0.016, *r*^2^ = 0.28).

## Discussion

Our current study demonstrated significant stereo deficits in PD, a longer response time with eye gazing, and less accurate saccades and fixations when evaluating these visual functions with easy to use, non-invasive methods, namely the 3D active shuttered system and Tobii eye tracker. The abnormalities in both stereopsis and oculomotor behavior were linked to the severity of the motor symptoms and cognitive status. One main focus is the application of stereo function as a possible marker in PD motor function. These tests are not routinely done in clinical settings. The widely used methodology in assessing stereopsis also has certain limitations. We demonstrated that the software-based stereo assessment is easy to use and may be more sensitive to detect stereo dysfunction, and such dysfunction is correlated to PD clinical features, i.e., motor and cognitive status.

Stereopsis is a complex process, which is dependent on an initial encoding of disparity in the striate cortex (Cumming and DeAngelis, 2001; Westheimer, 2009). Patients with PD with abnormal stereopsis demonstrated non-dominant extrastriate cortical atrophy (Koh et al., 2013). It is known that sensory integration is abnormal in PD. Several dopamine pathways are present in the human brain. Aside from the known deficits in the striatonigral pathway in PD individuals, the same has been seen in the ventral tegmental pathway and additional pathways that connect the ventral tegmentum to the amygdala, hippocampus, cingulate gyrus, and olfactory bulb (Weil et al., 2016; Armstrong, 2017). There is also significantly less activity in the visual cortex of people with PD (Nguyen-Legros, 1988; Harnois and Di Paolo, 1990). In addition, cerebral metabolic rates for glucose are decreased by up to 23% in the primary visual cortex of patients with PD (Eberling et al., 1994). There is a dopamine deficit in the retina and other structures in the peripheral visual system that can weaken visual function, which is related to poor stereopsis (Botha and Carr, 2012). For instance, a previous study has demonstrated that the peripheral visual pathway (i.e., color perception) can contribute to the stereo deficit (Sun et al., 2014). However, in our study design, we excluded individuals with abnormal visual acuity and contrast acuity, and with the screen visual tests, there were no differences between the control and PD groups. These tests are within the normal range, suggesting relatively intact peripheral afferent pathways. Therefore, the observed stereo deficit is most likely central in origin.

The basal ganglia are considered as a “sensory analyzer” engaged in central somatosensory control. The interconnections between the cortex, the basal ganglia, and the thalamus form an indirect basal ganglia-sensory loop (Boecker et al., 1999). Multiple sensory perception domains are abnormal in PD (Jobst et al., 1997; Cury et al., 2016; Hwang et al., 2016; Lester et al., 2017). Visual perception deficit is known to interfere with PD patients’ motor function, and the impairment in depth perception has a profound impact on patients’ navigation, mobility, and daily activities (Trick et al., 1994; Maschke et al., 2006; Kim et al., 2011). Therefore, it is necessary to consider clinical testing of the stereoscopic abilities of patients with PD, given how it impacts an individual’s functional status. In clinical practice, stereopsis is neither routinely assessed nor addressed in this population although patients may report visual deficits on widely used clinical questionnaires, i.e., Parkinson’s Non-motor Symptoms Questionnaire (Chaudhuri et al., 2006). To date, methods for stereo assessment include 2D imaging estimates, verbal estimates of depth perception (Ehgoetz Martens et al., 2013), Titmus stereotest (Kim et al., 2011; Sun et al., 2014), stereopsis questionnaires when recalling a video clip on a 3D TV (Lee et al., 2015), randomly placed dots that move as orthographic projections of a sphere on a screen (White et al., 2017), and a recording of the visually evoked potential associated with different 2D and 3D stimuli (Severac Cauquil et al., 2017). There are limitations with these methodologies, e.g., the main monocular cues, as well as the oculomotor function are not considered with the most widely used Titmus stereotest. The current study focused on stereopsis functions in patients with PD using the software-based 3D TV active shutter system, considering the monocular function and eye movement abnormalities. The active shutter system was proven to be more sensitive in detecting stereo deficits, especially subtler deficits in stereo perception in PD (Figure 2). We noticed that when the disparity was below the threshold of normal acuity (63 arc seconds, corresponding to the detail size on a 20/20 Snellen chart), there was a steeper decrement of performance in patients with PD. The stereopsis deficit was negatively correlated with LEDD. In addition, the degree of stereopsis change showed a trend of negative correlation with disease severity in PD, as reflected by UPDRS-III in our study. The relationship between motor performance and stereopsis was previously reported by Sun et al.–patients with PD with decreased stereopsis had a higher UPDRS-III score and worse motor function (Sun et al., 2014), consistent with our observations.

In PD, the cognitive function has been shown to correlate with performance in visual tasks. Patients with higher Mattis attention subscores performed better, and those who were able to maintain more steady attention during the visual discrimination task obtained logically better performances (Severac Cauquil et al., 2017). A more recent study with the stereopsis questionnaire and Titmus stereotest compared HCs with PD and patients with Alzheimer’s disease, and revealed no differences in the Titmus stereotest results among groups; however, in patients with PD, cognitive functions (MoCA scores) were correlated with the scores of the stereopsis questionnaire (Lee et al., 2015). Although patients with PD in that study have poor cognitive performance compared with our PD cohort, the results are similar to our observations. Therefore, our findings and previous reports on stereopsis abnormalities in PD and the associated cognitive decline may indicate behavioral complications of PD and the progression of the disease. As such, the presence of stereopsis dysfunction might serve as a clinical biomarker of PD to predict disease progression and outcomes.

Reductions in dopamine levels in the basal ganglia and frontal cortex also contribute to higher level oculomotor movements (Hikosaka et al., 2000). In our study with the eye-tracking system, we observed a prolonged visual response time in patients with PD compared to the control subjects. The visual response is an important component of information processing as it indexes the speed of stimulus processing and response programming (Rao et al., 1985). It connects visual scanning to movement reaction, which is considered a sensitive variable for measuring information processing of the oculomotion pathway. Patients with PD showed disturbed default mode network connectivity with the brain regions that are involved in visual processing and attentional network (Rektorova et al., 2014). In addition, the reconciliation of visual disparity with vergence and saccade movements is an essential step to form a cohesive 3D vision. In PD, saccade and vergence eye movements are reported to be abnormal (Gupta et al., 2021), interfering with the perception of depth and dimensionality critical for normal balance control and prevention of falls (Urwyler et al., 2014). Although vergence is not directly assessed in this study, it is known that under normal circumstances, the abrupt changes in the 3D gaze shift occur with the congruent movements of vergence and saccade (Erkelens et al., 1989; Zee et al., 1992; Rambold et al., 2002); and in PD, the gaze shifting strategy of the two eyes was incongruent (Gupta et al., 2021).

Stereopsis and visual response time are important in dynamic situations, which require rapid visual functions (Bauer et al., 2001). To date, there is still insufficient knowledge on how visual-locomotion coupling in patients with PD changes as the disease progresses. We found an impaired saccade movement and fixation. A very few studies that tested stereopsis performance can provide a combined analysis of stereo acuity while assessing ocular trajectory and response time at the same time, such an observation is a strength of our current study. Our observations in the link between visual behavior and motor and cognitive deficits suggest that such visual parameters can potentially serve as the clinical markers in PD when combined with other clinical, biochemical, and imaging biomarkers.

Although we did not observe significant differences in the average saccade amplitude or the total number of saccades between the control and PD groups, the saccadic movements were abnormal and manifested as more peaks and unstable fixations in patients with PD, which are consistent with the known characteristic multistep sequence of eye movements, namely the discrete small saccades in PD (DeJong and Jones, 1971).

There are a few limitations to this study. This is a single centered study with relatively small sample size. We did not have any *de novo* patients with PD as we are a tertiary referral center. We did not assess whether there is a difference in the visual behaviors during the OFF and ON medication states. All tests were done during the ON state. We did not test vergence directly—however, the saccade and fixation abnormality may indicate a deficit in vergence, which also impacts stereopsis. In the future, we plan to recruit more patients with further testing during both OFF and ON states to determine whether stereopsis and oculomotor behaviors can predict the response to treatments. We also plan to continue with a prospective study to observe how these visual functions evolve as disease progresses. In addition, we used 60 Hz as a sampling rate in our study, which is relatively low. Leube et al. (2017) compared eye trackers at 60 and 120 Hz with a 1,000-Hz eye tracker in a reading task. The saccade detection was generally adequate at 60 and 120 Hz, while the 120-Hz eye tracker was better in measuring the duration of the saccade than that of the 60 Hz. The number and duration of fixations were not significantly different between 60 and 120 Hz frequencies (Leube et al., 2017). While 120 Hz sampling results in higher accuracy in the detection of fast eye movements and fixation durations, which is necessary for a reading task. However, the design of our study is different. In contrast to the reading task, our study was testing initiation and velocity of saccade from one target to the next, to compare the PD individuals with age-matched controls. Such a task does not require the same high sampling rate as in reading. The differences observed at 60 Hz are still valid.

In conclusion, with the combination of the software-based 3D shutter system with oculomotor assessment, the current study revealed that patients with PD exhibit poorer stereopsis and impaired visual response time. The degree of stereo perception and visual response deficits reflected the clinical characteristics of PD. Our study suggested a potential role of testing these visual functions in patients with PD and also indicated stereo deficits and the possibility of impaired response time in PD to potentially serve as clinical biomarkers in PD research.

## Data Availability Statement

The raw data supporting the conclusions of this article will be made available by the authors, without undue reservation.

## Ethics Statement

The studies involving human participants were reviewed and approved by the University of Alberta Ethics Review Board. The patients/participants provided their written informed consent to participate in this study.

## Author Contributions

FB: research project – conception and organization, statistical analysis – design, execution, and review and critique, and manuscript – wrote the first draft and review and critique. TS: research project – organization and execution, statistical analysis – design and execution, and manuscript – wrote the first draft and review and critique. WH: research project – execution, statistical analysis – execution and review and critique, and manuscript – review and critique. JF and EM: research project – execution and manuscript – review and critique. BZ: research project – conception and organization, and manuscript – review and critique. All authors contributed to the article and approved the submitted version.

## Conflict of Interest

The authors declare that the research was conducted in the absence of any commercial or financial relationships that could be construed as a potential conflict of interest.

## Publisher’s Note

All claims expressed in this article are solely those of the authors and do not necessarily represent those of their affiliated organizations, or those of the publisher, the editors and the reviewers. Any product that may be evaluated in this article, or claim that may be made by its manufacturer, is not guaranteed or endorsed by the publisher.

## Supplementary Material

Supplementary Figure 1 Screening stereopsis with 2D images. In the 2D pictures, two or three objects of uniform size were shown that are distributed in depth. The study participants were asked to provide a cue to the depth judging the relative sizes of the objects.

## References

[B1] Alhassan M. Hovis J. K. Almeida Q. J. (2020). Stereopsis and ocular alignment in Parkinson’s disease patients with and without freezing of gait symptoms. *Clin. Exp. Opt.* 103 513–519. 10.1111/cxo.12961 31441118

[B2] Antoniades C. A. Bogacz R. Kennard C. FitzGerald J. J. Aziz T. Green A. L. (2014). Deep brain stimulation abolishes slowing of reactions to unlikely stimuli. *J. Neurosci.* 34 10844–10852. 10.1523/JNEUROSCI.1065-14.2014 25122887 PMC4131008

[B3] Armstrong R. A. (2011). Visual symptoms in Parkinson’s disease. *Parkinsons Dis.* 2011:908306. 10.4061/2011/908306 21687773 PMC3109513

[B4] Armstrong R. A. (2017). Visual dysfunction in Parkinson’s Disease. *Int. Rev. Neurobiol.* 134 921–946. 10.1016/bs.irn.2017.04.007 28805589

[B5] Bauer A. Dietz K. Kolling G. Hart W. Schiefer U. (2001). The relevance of stereopsis for motorists: a pilot study. *Graefes Arch. Clin. Exp. Ophthalmol.* 239 400–406. 10.1007/s004170100273 11561786

[B6] Boecker H. Ceballos-Baumann A. Bartenstein P. Weindl A. Siebner H. R. Fassbender T. (1999). Sensory processing in Parkinson’s and Huntington’s disease: investigations with 3D H(2)(15)O-PET. *Brain* 122(Pt 9), 1651–1665. 10.1093/brain/122.9.1651 10468505

[B7] Botha H. Carr J. (2012). Attention and visual dysfunction in Parkinson’s disease. *Parkinsonism Relat. Disord.* 18 742–747. 10.1016/j.parkreldis.2012.03.004 22503538

[B8] Chan F. Armstrong I. T. Pari G. Riopelle R. J. Munoz D. P. (2005). Deficits in saccadic eye-movement control in Parkinson’s disease. *Neuropsychologia* 43 784–796. 10.1016/j.neuropsychologia.2004.06.026 15721191

[B9] Chaudhuri K. R. Martinez-Martin P. Schapira A. H. Stocchi F. Sethi K. Odin P. (2006). International multicenter pilot study of the first comprehensive self-completed nonmotor symptoms questionnaire for Parkinson’s disease: the NMSQuest study. *Mov. Disord.* 21 916–923. 10.1002/mds.20844 16547944

[B10] Cumming B. G. DeAngelis G. C. (2001). The physiology of stereopsis. *Annu. Rev. Neurosci.* 24 203–238. 10.1146/annurev.neuro.24.1.203 11283310

[B11] Cury R. G. Galhardoni R. Fonoff E. T. Perez Lloret S. Dos Santos Ghilardi M. G. Barbosa E. R. (2016). Sensory abnormalities and pain in Parkinson disease and its modulation by treatment of motor symptoms. *Eur. J. Pain* 20 151–165. 10.1002/ejp.745 26147660

[B12] DeJong J. D. Jones G. M. (1971). Akinesia, hypokinesia, and bradykinesia in the oculomotor system of patients with Parkinson’s disease. *Exp. Neurol.* 32 58–68. 10.1016/0014-4886(71)90165-8 5095610

[B13] Eberling J. L. Richardson B. C. Reed B. R. Wolfe N. Jagust W. J. (1994). Cortical glucose metabolism in Parkinson’s disease without dementia. *Neurobiol. Aging* 15 329–335. 10.1016/0197-4580(94)90028-0 7936057

[B14] Ehgoetz Martens K. A. Ellard C. G. Almeida Q. J. (2013). Dopaminergic contributions to distance estimation in Parkinson’s disease: a sensory-perceptual deficit? *Neuropsychologia* 51 1426–1434. 10.1016/j.neuropsychologia.2013.04.015 23643554

[B15] Eidolab (2014). *EidoLab.* Available online at: http://eidomatica.di.unimi.it/index.php/research/stereo/stereotest (accessed August, 2018).

[B16] Erkelens C. J. Van der Steen J. Steinman R. M. Collewijn H. (1989). Ocular vergence under natural conditions. I. Continuous changes of target distance along the median plane. *Proc. R. Soc. Lond. B Biol. Sci.* 236 417–440. 10.1098/rspb.1989.0030 2567519

[B17] Fawcett A. P. Gonzalez E. G. Moro E. Steinbach M. J. Lozano A. M. Hutchison W. D. (2010). Subthalamic nucleus deep brain stimulation improves saccades in Parkinson’s disease. *Neuromodulation* 13 17–25. 10.1111/j.1525-1403.2009.00246.x 21992760

[B18] Gadia D. Garipoli G. Bonanomi C. Albani L. Rizzi A. (2014). Assessing stereo blindness and stereo acuity on digital displays. *Displays* 35 206–212. 10.1016/j.displa.2014.05.010

[B19] Garmham L. Sloper J. (2006). Effect of age on adult stereoacuity as measured by different type of stereotests. *Br. J. Ophthal.* 90 91–95. 10.1136/bjo.2005.077719 16361675 PMC1856927

[B20] Gupta P. Beylergil S. Murray J. Kilbane C. Ghasia F. F. Shaikh A. G. (2021). Computational models to delineate 3D gaze-shift strategies in Parkinson’s disease. *J. Neural. Eng.* 18:0460a5. 10.1088/1741-2552/ac123e 34233315 PMC8863489

[B21] Harnois C. Di Paolo T. (1990). Decreased dopamine in the retinas of patients with Parkinson’s disease. *Invest. Ophthalmol. Vis. Sci.* 31 2473–2475. 2243012

[B22] Heron S. Lages M. (2012). Screening and sampling in studies of binocular vision. *Vis. Res.* 62 228–234. 10.1016/j.visres.2012.04.012 22560956

[B23] Hikosaka O. Takikawa Y. Kawagoe R. (2000). Role of the basal ganglia in the control of purposive saccadic eye movements. *Physiol. Rev.* 80 953–978. 10.1152/physrev.2000.80.3.953 10893428

[B24] Hughes A. J. Daniel S. E. Ben-Shlomo Y. Lees A. J. (2002). The accuracy of diagnosis of parkinsonian syndromes in a specialist movement disorder service. *Brain* 125(Pt 4), 861–870. 10.1093/brain/awf080 11912118

[B25] Hwang S. Agada P. Grill S. Kiemel T. Jeka J. J. (2016). A central processing sensory deficit with Parkinson’s disease. *Exp. Brain Res.* 234 2369–2379. 10.1007/s00221-016-4642-4 27059036 PMC4925186

[B26] Jobst E. E. Melnick M. E. Byl N. N. Dowling G. A. Aminoff M. J. (1997). Sensory perception in Parkinson disease. *Arch. Neurol.* 54 450–454. 10.1001/archneur.1997.00550160080020 9109747

[B27] Kim S. H. Park J. H. Kim Y. H. Koh S. B. (2011). Stereopsis in drug naive Parkinson’s disease patients. *Can. J. Neurol. Sci.* 38 299–302. 10.1017/s0317167100011501 21320837

[B28] Koh S. B. Suh S. I. Kim S. H. Kim J. H. (2013). Stereopsis and extrastriate cortical atrophy in Parkinson’s disease: a voxel-based morphometric study. *Neuroreport* 24 229–232. 10.1097/WNR.0b013e32835edbc5 23376833

[B29] Lee C. N. Ko D. Suh Y. W. Park K. W. (2015). Cognitive functions and stereopsis in patients with Parkinson’s disease and Alzheimer’s disease using 3-dimensional television: a case controlled trial. *PLoS One* 10:e0123229. 10.1371/journal.pone.0123229 25822839 PMC4378891

[B30] Lester M. E. Cavanaugh J. T. Foreman K. B. Shaffer S. W. Marcus R. Dibble L. E. (2017). Adaptation of postural recovery responses to a vestibular sensory illusion in individuals with Parkinson disease and healthy controls. *Clin. Biomech.* 48 73–79. 10.1016/j.clinbiomech.2017.07.008 28783491

[B31] Leube A. Rifai K. Rifai K. (2017). Sampling rate influences saccade detection in mobile eye tracking of a reading task. *J. Eye Mov. Res.* 10:10.16910/jemr.10.3.3. 10.16910/jemr.10.3.3 33828659 PMC7141092

[B32] Li B. Xu D. Feng S. Wu A. Yang X. (2006). *Perceptual Depth Estimation from a Single 2D Image Based on Visual Perception Theory.* Berlin: Springer.

[B33] Maschke M. Gomez C. M. Tuite P. J. Pickett K. Konczak J. (2006). Depth perception in cerebellar and basal ganglia disease. *Exp. Brain Res.* 175 165–176. 10.1007/s00221-006-0535-2 16733701

[B34] Micieli G. Tassorelli C. Martignoni E. Pacchetti C. Bruggi P. Magri M. (1991). Disordered pupil reactivity in Parkinson’s disease. *Clin. Auton. Res.* 1 55–58. 10.1007/bf01826058 1821667

[B35] Momeni-Moghadam H. Kundart J. Ehsani M. Gholami K. (2011). *The Comparison of Stereopsis with TNO and Titmus Tests in Symptomatic and Asymptomatic University Students.* Boston, FL: American Academy of Optometry Annual Meeting.

[B36] Natsopoulos D. Bostantzopoulou M. S. Katsarou Z. Grouios G. Mentenopoulos G. (1993). Space deficits in Parkinson’s disease patients: quantitative or qualitative differences from normal controls? *Behav. Neurol.* 6 193–206. 10.3233/BEN-1993-6404 24487135

[B37] Nguyen-Legros J. (1988). Functional neuroarchitecture of the retina: hypothesis on the dysfunction of retinal dopaminergic circuitry in Parkinson’s disease. *Surg. Radiol. Anat.* 10 137–144. 10.1007/bf02307822 3135618

[B38] Nilsson M. H. Patel M. Rehncrona S. Magnusson M. Fransson P. A. (2013). Subthalamic deep brain stimulation improves smooth pursuit and saccade performance in patients with Parkinson’s disease. *J. Neuroeng. Rehabil.* 10:33. 10.1186/1743-0003-10-33 23551890 PMC3621588

[B39] Perkins J. E. Janzen A. Bernhard F. P. Wilhelm K. Brien D. C. Huang J. (2021). Saccade, pupil, and blink responses in rapid eye movement sleep behavior disorder. *Mov. Disord.* 36 1720–1726. 10.1002/mds.28585 33754406 PMC8359943

[B40] Pinkhardt E. H. Kassubek J. (2011). Ocular motor abnormalities in Parkinsonian syndromes. *Parkinsonism Relat. Disord.* 17 223–230. 10.1016/j.parkreldis.2010.08.004 20801069

[B41] Rambold H. Sprenger A. Helmchen C. (2002). Effects of voluntary blinks on saccades, vergence eye movements, and saccade-vergence interactions in humans. *J. Neurophysiol.* 88 1220–1233. 10.1152/jn.2002.88.3.1220 12205143

[B42] Rao S. L. Gangadhar B. N. Keshavan M. S. Hegde A. S. Nardev G. (1985). Reaction time deficits in post traumatic syndrome. *Indian J. Psychiatry* 27 63–65. 21927069 PMC3011169

[B43] Rektorova I. Krajcovicova L. Marecek R. Novakova M. Mikl M. (2014). Default mode network connectivity patterns associated with visual processing at different stages of Parkinson’s disease. *J. Alzheimers Dis.* 42(Suppl. 3), S217–S228. 10.3233/JAD-132684 25114077

[B44] Severac Cauquil A. Ory-Magne F. Jardine V. Galitzky M. Rosito M. Brefel-Courbon C. (2017). Parkinson’s patients can rely on perspective cues to perceive 3D space. *Brain Res.* 1663 161–165. 10.1016/j.brainres.2017.03.017 28327351

[B45] Sun L. Zhang H. Gu Z. Cao M. Li D. Chan P. (2014). Stereopsis impairment is associated with decreased color perception and worse motor performance in Parkinson’s disease. *Eur. J. Med. Res.* 19:29. 10.1186/2047-783X-19-29 24886673 PMC4046158

[B46] Sweeney J. A. Luna B. Srinivasagam N. M. Keshavan M. S. Schooler N. R. Haas G. L. (1998). Eye tracking abnormalities in schizophrenia: evidence for dysfunction in the frontal eye fields. *Biol. Psychiatry* 44 698–708. 10.1016/s0006-3223(98)00035-3 9798073

[B47] Tomlinson C. L. Stowe R. Patel S. Rick C. Gray R. Clarke C. E. (2010). Systematic review of levodopa dose equivalency reporting in Parkinson’s disease. *Mov. Disord.* 25 2649–2653. 10.1002/mds.23429 21069833

[B48] Trick G. L. Kaskie B. Steinman S. B. (1994). Visual impairment in Parkinson’s disease: deficits in orientation and motion discrimination. *Optom. Vis. Sci/* 71 242–245. 10.1097/00006324-199404000-00002 8047335

[B49] Tseng P. H. Cameron I. G. Pari G. Reynolds J. N. Munoz D. P. Itti L. (2013). High-throughput classification of clinical populations from natural viewing eye movements. *J. Neurol.* 260 275–284. 10.1007/s00415-012-6631-2 22926163

[B50] Urwyler P. Nef T. Killen A. Collerton D. Thomas A. Burn D. (2014). Visual complaints and visual hallucinations in Parkinson’s disease. *Parkinsonism Relat. Disord.* 20 318–322. 10.1016/j.parkreldis.2013.12.009 24405755

[B51] Vishwanath D. Kowler E. (2004). Saccadic localization in the presence of cues to three-dimensional shape. *J. Vis.* 4 445–458. 10.1167/4.6.4 15330712

[B52] Wang C. A. McInnis H. Brien D. C. Pari G. Munoz D. P. (2016). Disruption of pupil size modulation correlates with voluntary motor preparation deficits in Parkinson’s disease. *Neuropsychologia* 80 176–184. 10.1016/j.neuropsychologia.2015.11.019 26631540

[B53] Weil R. S. Schrag A. E. Warren J. D. Crutch S. J. Lees A. J. Morris H. R. (2016). Visual dysfunction in Parkinson’s disease. *Brain* 139 2827–2843. 10.1093/brain/aww175 27412389 PMC5091042

[B54] Westheimer G. (2009). The third dimension in the primary visual cortex. *J. Physiol.* 587(Pt 12), 2807–2816. 10.1113/jphysiol.2009.170175 19525565 PMC2718240

[B55] Westheimer G. (2013). Clinical evaluation of stereopsis. *Vis. Res.* 90 38–42. 10.1016/j.visres.2012.10.005 23092634

[B56] Wexler M. Ouarti N. (2008). Depth affects where we look. *Curr. Biol.* 18 1872–1876. 10.1016/j.cub.2008.10.059 19062283

[B57] White K. D. Skidmore F. M. Hammond J. Heilman K. M. (2017). The visual kinetic depth effect is altered with Parkinson’s disease. *Parkinsonism Relat. Disord.* 37 97–100. 10.1016/j.parkreldis.2017.01.004 28169155

[B58] Young D. E. Wagenaar R. C. Lin C. C. Chou Y. H. Davidsdottir S. Saltzman E. (2010). Visuospatial perception and navigation in Parkinson’s disease. *Vis. Res.* 50 2495–2504. 10.1016/j.visres.2010.08.029 20837045 PMC3008343

[B59] Yugeta A. Terao Y. Fukuda H. Hikosaka O. Yokochi F. Okiyama R. (2010). Effects of STN stimulation on the initiation and inhibition of saccade in Parkinson disease. *Neurology* 74 743–748. 10.1212/WNL.0b013e3181d31e0b 20194913

[B60] Zee D. S. Fitzgibbon E. J. Optican L. M. (1992). Saccade-vergence interactions in humans. *J. Neurophysiol.* 68 1624–1641. 10.1152/jn.1992.68.5.1624 1479435

