# Supplementary figures and images for: Stereopsis and Eye Movement Abnormalities in Parkinson’s Disease and Their Clinical Implications
Source: Front Aging Neurosci. 2022 Feb 8;14:783773. doi: 10.3389/fnagi.2022.783773 (PMC8861359; doi:10.3389/fnagi.2022.783773)

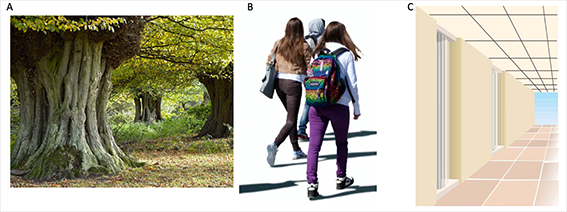

Supplement: Supplementary Figure 1 — Screening stereopsis with 2D images. In the 2D pictures, two or three objects of uniform size were shown that are distributed in depth. The study participants were asked to provide a cue to the depth judging the relative sizes of the objects. [file Image_1.TIFF]
